# A National Comparative Investigation of Twins With Congenital Heart Defects for Neurodevelopmental Outcomes and Quality of Life (Same Same, but Different?): Protocol for a Prospective Observational Study

**DOI:** 10.2196/26404

**Published:** 2021-05-13

**Authors:** Julia Remmele, Paul Christian Helm, Renate Oberhoffer-Fritz, Ulrike MM Bauer, Thomas Pickardt, Peter Ewert, Oktay Tutarel

**Affiliations:** 1 Department of Congenital Heart Defects and Pediatric Cardiology German Heart Center of Munich Munich Germany; 2 Institute of Preventive Pediatrics Technical University Munich Munich Germany; 3 National Register for Congenital Heart Defects German Center for Cardiovascular Research (DZHK) Berlin Germany; 4 Partner Site Munich Heart Alliance German Center for Cardiovascular Research (DZHK) Munich Germany

**Keywords:** congenital heart defect, twin siblings with CHD, twin study, neurodevelopmental outcome, same same, cardiology, heart defect, twin

## Abstract

**Background:**

Due to the increased survival rates of patients with congenital heart defects (CHD), associated disorders are an increasing focus of research. Existing studies figured out an association between CHD and its treatment, and neurodevelopmental outcomes including motor competence impairments. All these studies, however, compared their test results with reference values or results of healthy control groups. This comparison is influenced by socioeconomic and genetic aspects, which do have a known impact on neurodevelopmental outcomes.

**Objective:**

This study protocol describes a setting that aims to find out the role of CHD and its treatments on neurodevelopmental outcomes, excluding socioeconomic and genetic aspects. Only a twin comparison provides the possibility to exclude these confounding factors.

**Methods:**

In a German-wide prospective cohort study, 129 twin siblings registered in the National Register for Congenital Heart Defects will undergo testing on cognitive function (Wechsler Intelligence Tests age-dependent: Wechsler Adult Intelligence Scale, fourth edition; Wechsler Intelligence Scale for Children, fifth edition; and Wechsler Preschool and Primary Scale of Intelligence, fourth edition) and motor competence (Movement Assessment Battery for Children, second edition). Additionally, the self-reported health-related quality of life (KINDL-R for children, Short Form 36 for adults) and the parent-reported strength and difficulties of the children (Strength and Difficulties Questionnaire, German version) will be assessed by standardized questionnaires. CHD data on the specific diagnosis, surgeries, transcatheter procedures, and additional medical information will be received from patient records.

**Results:**

The approval of the Medical Ethics Committee Charité Mitte was obtained in June 2018. After getting funded in April 2019, the first enrollment was in August 2019. The study is still ongoing until June 2022. Final results are expected in 2022.

**Conclusions:**

This study protocol provides an overview of the study design’s technical details, offering an option to exclude confounding factors on neurodevelopmental outcomes in patients with CHD. This will enable a specific analysis focusing on CHD and clinical treatments to differentiate in terms of neurodevelopmental outcomes of patients with CHD compared to twin siblings with healthy hearts. Finally, we aim to clearly define what is important to prevent patients with CHD in terms of neurodevelopmental impairments to be able to develop targeted prevention strategies for patients with CHD.

**Trial Registration:**

German Clinical Trials Register DRKS00021087; https://tinyurl.com/2rdw8w67

**International Registered Report Identifier (IRRID):**

DERR1-10.2196/26404

## Introduction

Congenital heart defects (CHD) are the most common congenital malformation and are associated with increased morbidity and mortality [1,2]. Based on the medical progress made in recent decades in the fields of prenatal diagnostics, pediatric cardiology, and heart surgery, mortality has been substantially reduced, and life expectancy has increased significantly [1,3,4]. Therefore, currently, more than 90% of children with CHD reach adulthood [1,5-7]. Thus, a major scientific focus lies on the clinical outcome and especially on neurologic concomitant diseases or sequela. Newborns with a CHD are already considered to be at risk of often starting with acidosis and low Apgar levels after delivery. After birth, acute initial oxygen deficiency, low cardiac output, and cyanosis are risk factors as are medical interventions such as surgery, transcatheter interventions, or other invasive medical procedures that may influence the developing brain [8-10].

Several studies on patients with CHD after surgery have shown that neurodevelopment, including motor competence, is significantly impaired compared to healthy controls [9,11-14]. Although it seems obvious to consider the heart defect and its treatment consequences as the main cause of this difference, the patient’s genetic predisposition, individual support, and socioeconomic factors play a central role in cognitive development as well [15,16]; it is, however, not known to what extent. How would the same child have developed without the CHD? Theoretically, these influences could be differentiated, comparing patients with CHD with healthy volunteers who have the same genetic predisposition and the same socioeconomic environment. In a practical approximation, our study on twins of whom only one sibling has CHD tries to differentiate the influence of heart defects and medical treatment on one hand from genetic predisposition and environmental factors on the other hand, focusing on neurodevelopmental outcome.

## Methods

### Study

**Figure 1:**
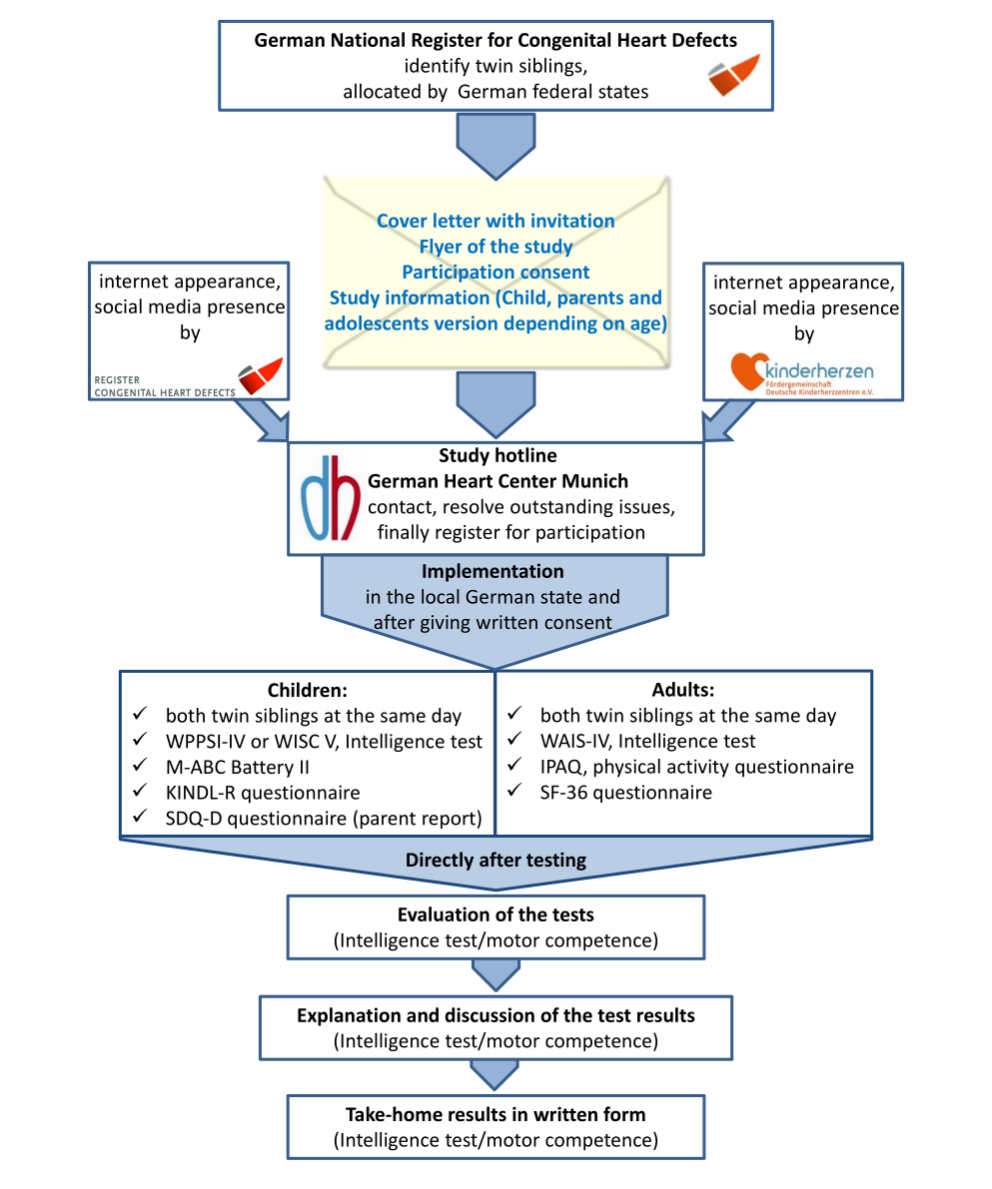
Flowchart of recruitment and implementation. IPAQ: International Physical Activity Questionnaire; M-ABC: Movement Assessment Battery for Children; SDQ: Strength and Difficulties Questionnaire, German version; SF-36: Short Form 36; WAIS-IV: Wechsler Adult Intelligence Scale, fourth edition; WISC V: Wechsler Intelligence Scale for Children, fifth edition; WPPSI-IV: Wechsler Preschool and Primary Scale of Intelligence, fourth edition.

This study is a national, German-wide prospective cohort study investigating twin siblings with at least one having a CHD. They are registered in the National Register for Congenital Heart Defects (NRCHD), the largest register for patients with CHD in Europe [17]. The inclusion takes place by written information sheets and an invitation to participate (see Figure 1). Participation in the study is voluntary and only takes place after the participants or, in the case of minors, their parents have given their written consent.

### Participants

The study population consists of patients with CHD and their twin siblings as well as both twins having CHD. To enable the participation of as many twins as possible, to keep the effort for the participants as low as possible, and to offer optimal test conditions with short travel distances and the same test settings, the tests are carried out at regional test facilities performed by one single investigator for all the tests throughout Germany.

### Inclusion Criteria

The inclusion criteria were the following: all kinds of CHD (this includes all cardiac diagnoses defined by the International Paediatric and Congenital Cardiac Code [18]), age between 3-99 years, and both twins or their parents agreeing to participate.

### Exclusion Criteria

The exclusion criteria were the following: surgery or interventional treatments within the last 6 month, massive mental retardation (to avoid a selection bias, all patients who wish to participate are admitted; if testing is not possible due to massive mental retardation, the twin siblings are excluded from the analysis but recorded as “drop-outs”), other medical examinations on the test day, or insufficient language skills (German).

### Procedure

#### Primary Outcome

##### Wechsler Intelligence Test

The Wechsler Intelligence Test is designed for three age groups to assess cognitive function. The current version of the Wechsler Preschool and Primary Scale of Intelligence, fourth edition [19] is used for children aged 3-7 years, and the Wechsler Intelligence Scale for Children, fifth edition [20] is intended for children and young people aged 6-16 years. Finally, the Wechsler Adult Intelligence Scale, fourth edition [21] is an intelligence test for adolescents and adults within an age range of 16-99 years.

These tests consist of 10 subtest groups, which results in IQs for the four competence areas (working memory IQ, verbal comprehension IQ, processing speed IQ, and perceptual logical thinking IQ) and a full-scale IQ calculated using the results of all subtest groups.

##### Motor Competence

For the evaluation of motor competence, the Movement Assessment Battery for Children, second edition (M-ABC 2) [22] is used. It is a standardized test for assessing the motor competence of children aged 3-16.9 years. The M-ABC 2 is divided into three competence groups according to age (first: 3-6 years; second: 7-10 years; third: 11-16 years) and thus adequately records the three competence categories: manual dexterity (consisting of 3 tests), ball skill (consisting of 2 tests), and balance (consisting of 3 tests).

The total test value, consisting of all three areas, represents motor competence [23].

##### International Physical Activity Questionnaire

To measure adult participants’ physical activities in everyday life, the International Physical Activity Questionnaire (IPAQ) [24] for adult patients will be used, due to there being no international standardized motor assessment battery for adults. The test results are categorized into three activity levels:

1. Health-promoting active (vigorous intensity activity on at least 3 days achieving a minimum of at least 1500 metabolic equivalent task [MET] minutes per week or 7 days of any combination of walking, moderate intensity, or vigorous intensity activities achieving a minimum of at least 3000 MET minutes per week)
2. Minimally active (3 or more days of vigorous activity of at least 20 minutes per day; 5 or more days of moderate intensity activity or walking of at least 30 minutes per day; or 5 or more days of any combination of walking, moderate intensity, or vigorous intensity activities achieving a minimum of at least 600 MET minutes per week)
3. Inactive (no activity reported or some activity reported but not enough to meet *health-enhancing physical active* or *minimally active*) [25]

The IPAQ is closely correlated with the results of spiroergometry [25].

#### Secondary Outcome

##### KINDL-R Questionnaire to Assess the Health-Related Quality of Life

To assess the health-related quality of life, parents (for preschool age children) and children receive the KINDL-R questionnaire [26], which they fill in independently. This is a multidimensional generic instrument for recording health-related quality of life. There are three versions for the corresponding age groups (first: 3-6 years; second: 7-12 years; third: 13-17 years); these comprise 24 questions, and validation has already been carried out [26].

##### Short Form 36 Questionnaire for Measuring Health-Related Quality of Life in Adults

The Short Form 36 (SF-36) consists of 36 questions and is a general health questionnaire that allows statements about the patient’s health status using means of 8 different dimensions [27]. It makes statements about general health perception (5 questions), physical health (10 questions), limited physical role function (4 questions), physical pain (2 questions), vitality (4 questions), mental health (5 questions), limited emotional role function (3 questions), and social functioning (2 questions).

The possible score ranges from 0 to 100 points. Zero points represent the worst quality of life value in terms of health, while 100 points describe the best possible state of health. Bullinger and Kirchberger [27] validated the German version, and the SF-36 is used to evaluate individual patients’ health status and monitor and compare disease burden with an acceptable internal consistency [28]. Therefore, it is used worldwide and is a well-established questionnaire, which is used in various fields of medicine, with great clinical relevance and is available in over 170 languages.

##### Strength and Difficulties Questionnaire

The Strength and Difficulties Questionnaire, German version (SDQ-D) [29] assesses behavioral problems and strengths in children and young people aged 4-17 years, and it is available in over 75 languages. The two-page parent-reported questionnaire contains a total of 25 characteristics, 10 of which are positive, 14 negative, and 1 neutral, and asks about problematic experiences of the child.

The SDQ-D measures the scales emotional problems, conduct problems, hyperactivity, behavioral problems with peers, and prosocial behavior.

From these scale scores, a total problem score is calculated, ranging in value from 0 to 40. Validation and updating of age-specific German reference values by Robert-Koch Institute published in 2020 [30].

### Data Handling

Since the study participants come from the NRCHD and the data processing takes place under the umbrella of the NRCHD, the study is subject to the data protection concept established in the NRCHD. All study participants already have a pseudonym and a randomly generated number as a result of their participation in the NRCHD. The latter is used to identify the questionnaires. The data obtained are stored separately from the personal identifying data under the aforementioned pseudonym. All information and data remain within the jurisdiction of the NRCHD. People outside this area, except for the study directors, have no access to the data. The study director conducts the tests personally.

The collection and storage of all data are carried out following the NRCHD’s data protection concept, which is registered with the Berlin Commissioner for Data Protection and Freedom of Information (No. 531.390). The study directors receive the data for statistical evaluation for a limited time and without direct reference to the participating persons. In addition, only NRCHD employees who are bound to secrecy have access to the data. The data collected via German Heart Center Munich are stored on hospital servers and only the research team has access. Data transfer between NRCHD and German Heart Center Munich takes place in person or a password-protected version. The written consent and collected data will be stored separately for 10 years after the end of the study. At the end of the study, both the participants and the funding agency will be informed about the results.

### Statistical Analysis

#### Power Analysis and Sample Size

Due to the explorative character of the study and the, so far, unknown prevalence of CHD in twin siblings in Germany or in any other country, this makes adequate case number planning difficult. Using G*Power analysis for “a priori required sample size” for student *t* test with paired samples with a medium effect size (0.5), a power set to 0.95, and an alpha error probability set to .05, we ended up with a total number of 54 twins. However, this study aims for a total survey of twins with CHD living in Germany. Based on previous experience, a conservative estimate of at least 50% inclusion can be expected, that is, 129 pairs of twins from 259 twins recorded in the NRCHD throughout Germany (as of February 2018).

### Primary and Secondary Analysis

**Figure 2:**
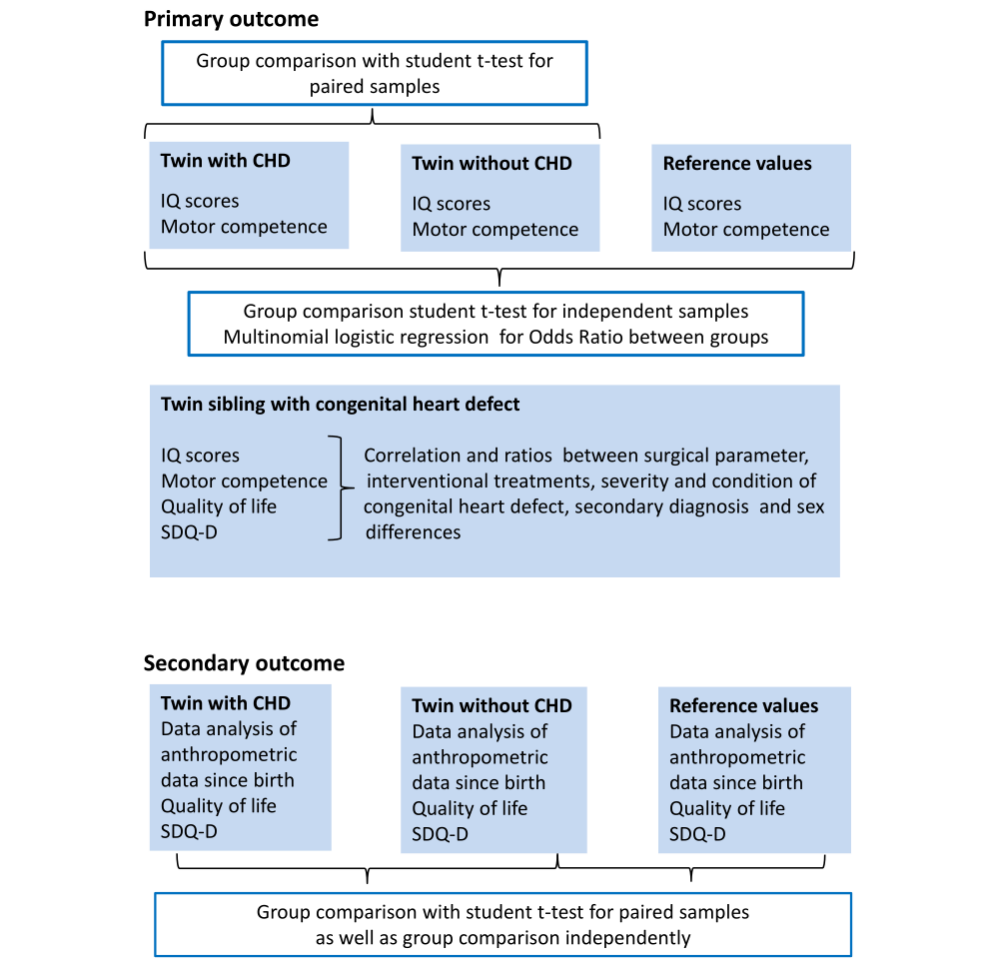
Planned statistical analysis. CHD: congenital heart defects; SDQ-D: Strength and Difficulties Questionnaire, German version.

The planned primary and secondary analysis are displayed in Figure 2.

## Results

The approval of the Medical Ethics Committee Charité Mitte was obtained on June 26, 2018 (EA2/086/18). After getting funded in April 2019, first enrollment began in August 2019. The study is still ongoing until June 2022. Final results are expected in 2022.

## Discussion

This study protocol provides an overview of technical details of the study design, offering an option to exclude confounding factors on neurodevelopmental outcomes in patients with CHD. This will enable a specific analysis focusing on CHD and clinical treatments to differentiate in terms of neurodevelopmental outcomes of patients with CHD compared to twin siblings with healthy hearts. In the end, we aim to clearly define what is important to prevent patients with CHD in terms of neurodevelopmental impairments and to define targeted prevention strategies for patients with CHD.

## Abbreviations

CHD: congenital heart defects
IPAQ: International Physical Activity Questionnaire
MET: metabolic equivalent task
M-ABC 2: Movement Assessment Battery for Children, second edition
NRCHD: National Register for Congenital Heart Defects
SDQ-D: Strength and Difficulties Questionnaire, German version
SF-36: Short Form 36
